# Collaboration in Complex Systems: Multilevel Network Analysis for Community-Based Obesity Prevention Interventions

**DOI:** 10.1038/s41598-019-47759-4

**Published:** 2019-08-29

**Authors:** Jaimie McGlashan, Kayla de la Haye, Peng Wang, Steven Allender

**Affiliations:** 1 Global Obesity Centre, Deakin University, Geelong, Australia; 2 Department of Preventive Medicine, University of Southern California, Los Angeles, California, United States of America; 3 Centre for Transformative Innovation, Swinburne University of Technology, Melbourne, Australia

**Keywords:** Public health, Risk factors, Disease prevention

## Abstract

Community-based systems interventions represent a promising, but complex approach to the prevention of childhood obesity. Existing studies suggest that the implementation of multiple actions by engaged community leaders (steering committees) is of critical importance to influence a complex system. This study explores two key components of systems interventions: (1) steering committees; and (2) causal loop diagrams (CLDs), used to map the complex community-level drivers of obesity. The interactions between two components create an entangled, complex process difficult to measure, and methods to analyse the dependencies between these two components in community-based systems interventions are limited. This study employs multilevel statistical models from social network analysis to explore the complex interdependencies between steering committee collaboration and their actions in the CLD. Steering committee members from two communities engaged in obesity prevention interventions reported on their collaborative relationships with each other, and where their actions are situated in a locally developed CLD. A multilevel exponential random graph model (MERGM) was developed for each community to explore the structural configurations of the collaboration network, actions in the CLD, and cross-level interactions. The models showed the tendency for reciprocated and transitive collaboration among committee members, as well as some evidence of more complex multilevel configurations that may indicate integrated solutions and collective action. The use of multilevel network analysis represents a step toward unpacking the complexities inherent in community-based systems interventions for obesity prevention.

## Introduction

Obesity is driven by many factors such as environmental characteristics, social processes, government policy, community infrastructure, and human behaviour. This complexity of proximal and distal cause and effect makes prevention difficult^1,2^. A major challenge to prevention is the difficulty in conceptualising the interactions between these factors to enable practitioners to identify the best places to target for effective prevention^3^. To date the majority of efforts have targeted single factors, and in the face of complexity this has proven ineffective^4^. Leaders in the field now call for new science to understand, share and engage with the complexity inherent in obesity to develop, implement and continually improve obesity prevention efforts and other complex problems in public health^5^.

Despite the limited success of obesity prevention efforts to date, there are some promising approaches. An effective approach to tackling the complexity of obesity seems to come from community-based interventions^6^ whereby community leaders band together to co-create actions with community leaders, practitioners, and other key stakeholders. Several of these community-based interventions have led to significant reductions in measured obesity prevalence and age standardized body mass index^7–9^. The implementation of community-based interventions relies on acknowledging the complexity of obesity, and on providing the means for key actors in a community to identify parts of the system on which they can intervene and sustainably change^10^. These ‘whole-of-system’ interventions represent the next step in the science of obesity prevention, and their explicit ethos of engaging with complexity creates new challenges for program design and evaluation^11^.

Retrospective analyses of successful community-based interventions for obesity prevention has suggested that the strength of the steering committees, which are typically comprised of local leaders and key stakeholders who collaborate to implement the intervention, was a key driver of the interventions’ effectiveness and sustainability^12,13^. Steering committee collaboration networks appear to be critical in the diffusion of information about community health risks and potential actions, and in engaging broader community members in systems interventions^14^.

Innovation in the field includes the application of social network analysis (SNA) to measure and quantitatively analyse the patterns of relationships (i.e., networks) among steering committee members, as a tool for intervention evaluation^15–17^. In a substance abuse prevention initiative that leveraged community coalitions, Valente *et al*. (2007) surveyed more than 400 community leaders nested in 24 locations, and mapped relationships of advice-seeking among leaders in each coalition. They found that a greater density of advice seeking among coalition members (meaning a greater proportion of members that sought advice from one another) *did not* lead to increased program adoption, as might have been expected. Rather, their results suggested that too much cohesion and communication could be counterproductive, and they cautioned that less cohesive coalitions may be more adept at adopting the programs and acquiring the resources needed to do so in community initiatives^17^.

An application of SNA in obesity prevention analysed multiple relationship features (closeness, influence, and discussion) among community steering committee members from two successful interventions^16^. In both intervention sites, influential relationships were found to be dense *within* the steering committee, and close relationships tended to be with community members external to the steering committee. The observed network structures seemed to be supportive diffusion of the intervention^16^.

Despite these advances, we do not yet understand how networks of collaboration among intervention steering committees are embedded in, and linked to, other factors in the ‘whole-of-system’ community-based interventions. Specifically, we lack insight into how the structure and function of networks among steering committee members interacts with the formal description of the complex system of community factors that are associated with obesity risk. An increasingly popular approach in the design of ‘whole-of-system’ intervention are conceptualizations of the system using a mapping technique to create causal loop diagrams (CLDs). In current trials^10,18^, these diagrams are developed by the steering committees through group model building workshops^19^, and provide maps to conceptualize the intricate, complex system of obesity drivers (variables) relevant to their local community. Steering committee members use the maps to assign themselves to actions, where they identify parts of the CLD they could change using their existing capacity^10^.

Although there has been work in the obesity prevention literature contributing to the evaluation of steering committee networks^15,16^ and the analysis of systems diagrams^20^, to date these have been analysed independently, and failed to capture the interplay between the collaboration networks and the complex system. Given the complexity inherent in community-based interventions, it is important to understand how the distribution of actions around the system influences collaborative patterns between steering committee members. Without understanding the potential interdependencies between steering committee networks and the broader system, we can only draw conclusions about these components in isolation, and in reality, this simplification is likely to be insufficient^21^ and may miss potential areas to strengthen collaboration and improve intervention outcomes.

There is growing evidence of the effectiveness of applying *multilevel network methods* in diverse fields to identify meaningful patterns of interdependencies across multiple levels of a system^22^, relative to what can be understood via simplified, single layer network analyses^23^. For example, Bodin *et al*. (2016) used multilevel networks to model collaboration on complex tasks in disaster management, and to identify deeper structural complexities than could be captured by only conceptualizing this problem as a single layer network^24^. The researchers sought to identify the structural patterns of collaboration among social actors, and their task interdependencies, that leads to more successful implementation of task performance^24^. Certain structures, such as cross-level closure (where actors who work on connected tasks collaborate), were found to be associated with greater task performance due to shared resources and knowledge^24^. These types of processes may also be beneficial formations in community-based obesity prevention interventions. Multilevel networks have also increased our understanding of complex social processes in settings such as schools^25^, organizations^26^ and research collaboration^27^.

Systems interventions in public health are complex and difficult to evaluate^11^, and recent calls have been made to acknowledge the great amounts of complexity, rather than considering single components independently^5^. In this study we propose the use of multilevel network analysis to explore the way in which steering committees in current public health interventions collaborate, and how this collaboration network is associated with the complex system on which they are acting.

**Figure 1:**
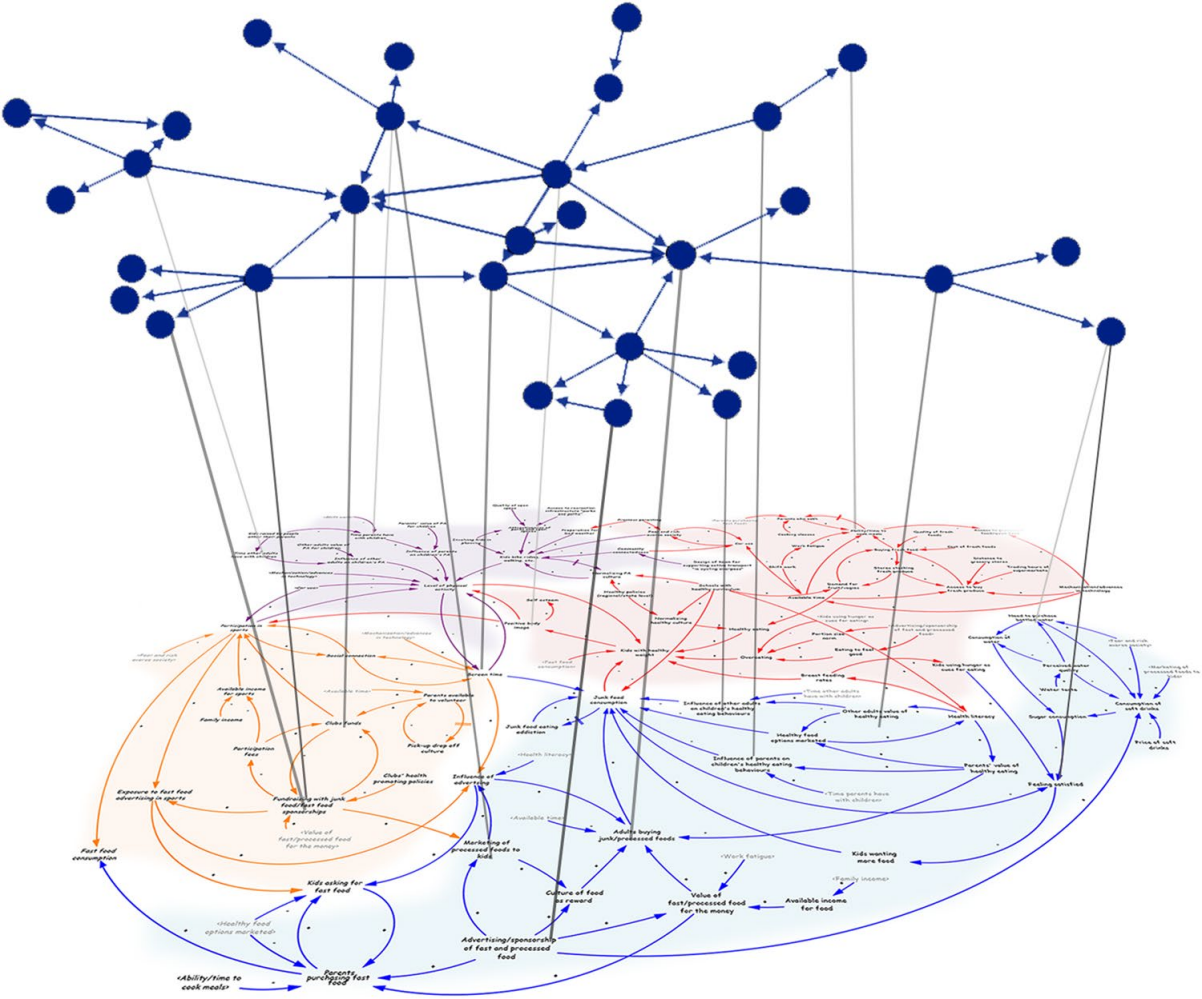
Conceptualization of a steering committee social network overlayed on the causal loop diagram to create a multilevel structure (not real data).

Using data from two communities engaged in whole community systems thinking trial for the prevention of childhood obesity^18^, we treat a locally developed causal loop diagrams as a network at one level that captures the interdependence of key drivers or factors that affect childhood obesity (called “CLD variables”); the collaboration network among steering committee members is at another level; and the “actions” by the community leaders on the CLD variables is defined as the *inter-level* network. A conceptual example of this construct is shown in Fig. 1, though this does not present the data captured in this paper. To our knowledge, this is the first multilevel network analysis that uses this approach to test for interrelationships and structural dependencies between these multilevel networks. Broadly, this study explores structural features of collaboration networks that may be associated with positive impacts on community level change to reduce obesity risks.

## Methods

### Setting

Two prospective community-based obesity prevention interventions were analysed in this research. Both aim to target childhood obesity prevention through multiple settings and actions, and are underpinned by systems thinking in their design. The interventions are each led by a separate highly engaged steering committee, and have followed an initial process developing CLDs specific to each community through group model building workshops^10^. These workshops involve the steering committees describing the dynamic factors that contribute to obesity in their community, along with their complex causal relationships, to ultimately build a CLD. The communities are located in western Victoria, Australia, and are a part of a larger state-wide intervention known as the ‘Whole of Systems Trial of Prevention Strategies for Childhood Obesity’ (WHO STOPS Childhood Obesity)^18^. At the time of data collection, Community 1 had been in progress for two years and Community 2 was approximately three years into the intervention.

### Participants

Participants were defined as those who are actively engaged in the steering committees and were identified with the assistance of local leaders from the Primary Care Partnership (PCP), an organization whose remit is to act as a platform to bridge local health service providers. There were 27 eligible steering committee members from Community 1 and 27 eligible steering committee members Community 2. Ethics clearance was received from the Deakin University Human Ethics Committee, and the study’s methods were implemented in accordance with the committee’s guidelines. Informed consent was provided by all individuals participating in the survey, and all participants were over 18 years of age.

### Data collection

A survey was conducted in December 2017 using Qualtrics to collect basic demographic information, including participant’s name, education level, gender, organizational affiliations (defined as their primary place of employment) and their roles at that organization. The organizational affiliation and role questions had a list of workplaces and associated roles common to the communities, with space for ‘Other’ responses. Organizations were grouped into ‘types’ for analysis including government, education, health service, primary care partnership, primary health network and other.

A social network section asked the participants to identify ‘*who they regularly collaborate with on issues related to healthy eating and physical activity environments within your community*’, from a list of other steering committee members. The number of people they could nominate was not restricted.

To identify steering committee member’s actions to variables in the CLD (i.e., committee members’ “actions” towards CLD variables), participants were shown the CLD developed by their community leaders (which, in most cases was a subset of the committee member participants), and were asked to ‘*select the areas of the CLD that you believe you have made an effort to improve*’ as part of their role with the intervention. This was presented to participants as an interactive CLD, and they were required to select specific obesity drivers (CLD variables), which changed colour when selected. Participants could select as many or few variables in the CLD as desired.

### Analytical methods

#### Defining the multilevel networks

**Figure 2:**
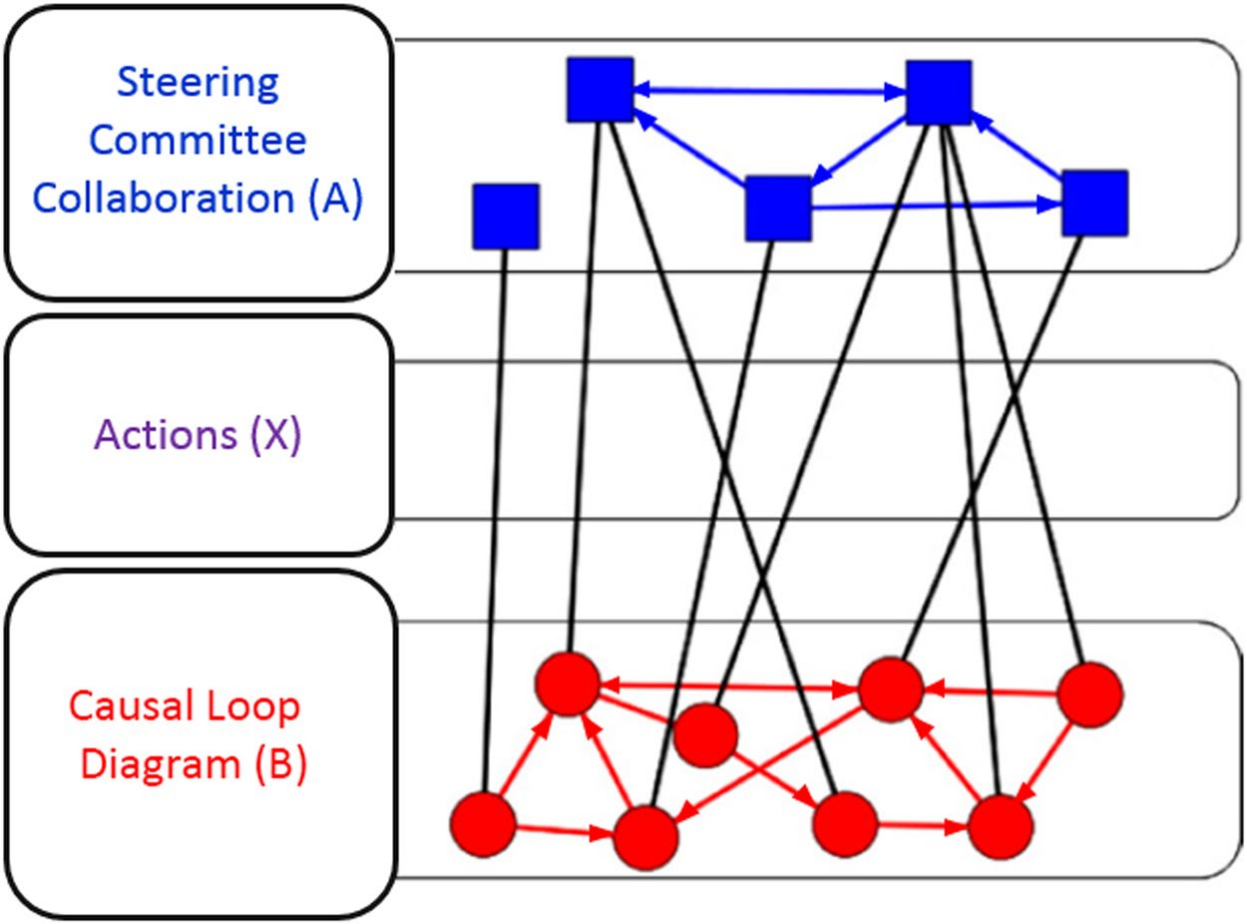
Directed multilevel network schematic diagram (adapted from Wang *et al*., 2013).

The primary dependent variables were treated as one multilevel network within each community, as illustrated in Fig. 2 (adapted from^28^), and comprised of: the collaboration network among steering committee members (network A); the community CLD (network B); and the links that represent actions from steering committee members towards the variables (nodes) in the CLD (network X). In this study, both network A and network B were treated as directed networks, meaning that a tie that is sent from node *i* to node *j* is defined independently from a tie sent from node *j* to node *i*.

We use the labels of the networks {*A*, *B*, *X*} as the network random variables. Each of the network variables comprised of a set of network tie random variables, the number of which depends on the number of nodes in each of the networks. For example, In the steering committee collaboration network (*A*) with *n* committee members, the network is a collection of *n*(*n* − 1) tie variables {*A*_*ij*_} where (*A*_*ij*_ = 1), if node *i* nominated node *j* as a collaboration partner, 0 otherwise. In the CLD (network B), a tie (*B*_*ij*_ = 1)), if node *i* was defined as having a direct causal effect on node *j* in the CLD^20^. Finally, “action” ties sent from steering committee members to obesity drivers in the CLD (network X) were defined as (*X*_*ij*_ = 1)) if the steering committee member, *i* reported that they were acting on CLD variable *j*. Network X is treated as a non-directed network, that is, *X*_*ij*_ = *X*_*ji*_.

#### Multilevel exponential random graph modelling

Exponential random graph models (ERGMs) provide a method for modelling the complex structure of social networks, and identifying the underlying social processes most likely to explain the observed structure. ERGMs are based on parameter estimates for endogenous network configurations and their interactions with exogenous node-level attributes^29^. ERGMs were initially developed for single layer networks^29^, but have been extended as *Multilevel ERGMs* (MERGMs) to assess network independencies across multiple layers of network data^28^.

Using the lower-case letter to represent realizations of our network variables, that is {*A*, *B*, *X*} = {*a*, *b*, *x*}; and (*Y*_*i*_ = *y*_*i*_) represent attribute variables for steering committee members in the collaboration network (*A*), such as their levels of education or affiliated organizations as categorical attributes.

The *multilevel ERGMs* (MERGMs) for our networks can be expressed as

$${P}_{\theta }(A=a,X=x|B=b,Y=y)=\frac{1}{\kappa }\exp \sum _{Q}{\theta }_{Q}{z}_{Q}(A,X,B,Y)$$

where we model the structure of collaboration network among steering committee members (*A*) and choices of actions (*X*), while treating the CLD network (*B*) and steering committee members’ attributes (*Y*) as exogenous, meaning they was fixed and used to explain (*A*) and (*X*).

**Figure 3:**
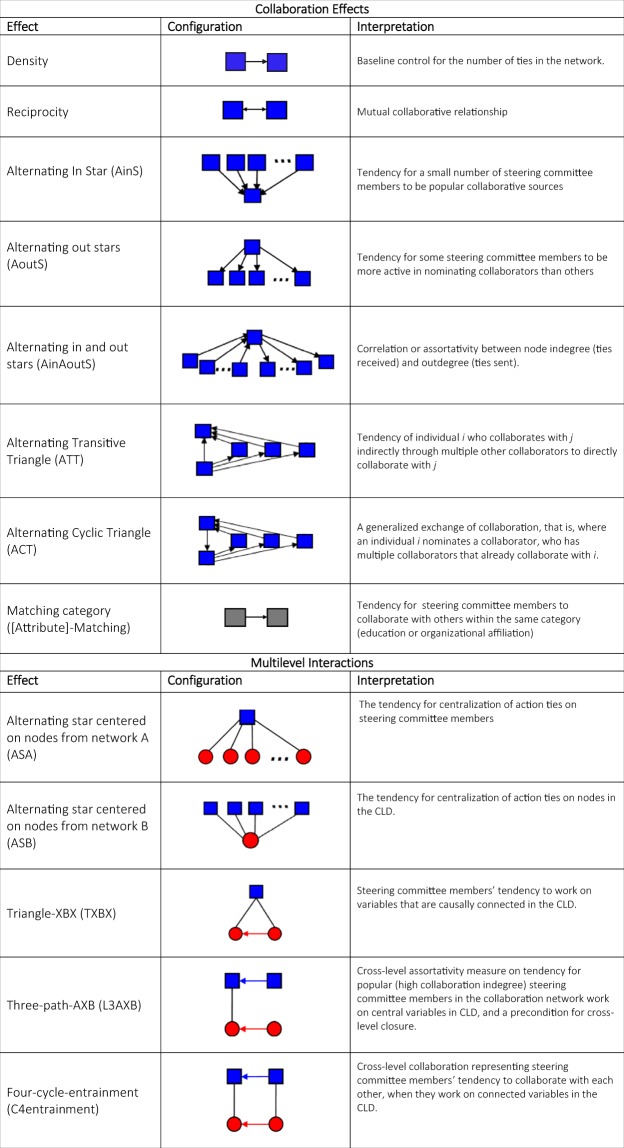
Selected MERGM configurations and possible interpretations. Note: blue nodes represent steering committee members, and red nodes represent CLD variables.

*Q* represents network configurations of type *Q* within which all tie variables and nodal attributes are considered as conditionally dependent following the hierarchy of tie dependence assumptions, that is, the occurrence of a tie is dependent on the existence of other network ties. These tie configurations or model effects can represent structural processes, e.g., the tendency to form reciprocal ties as a form of exchange, or interactions between network ties and node-level attributes, e.g., the preference for ties to be sent to nodes with a particular attribute. Figure 3 provides a list of selected MERGM configurations we used in our models with their visualization and potential interpretations.

z_Q_(*A*, *X*, *b*, *y*) are network statistics counting the number of configurations of type *Q*, which has the typical form of ${z}_{Q}(A,X,b,y)=\sum _{\{A,X\}}\,\prod _{(A,X,b,y)\in Q}\,AXby$. These tie configurations (model effects) can represent structural processes (e.g., reciprocity) or interactions between network ties and node-level attributes (e.g., preference for ties to be sent to nodes with a particular attribute).

*θ*_Q_ are parameters associated with configurations of type *Q*. A positive and significant estimate of *θ*_Q_ suggest the corresponding configurations happens more than one would expect from random given the rest of the model, while negative estimates mean the opposite. A parameter is considered significant if its estimate is more than twice the estimated standard error.

*κ* is a normalizing constant summing over the entire graph space bounded by the number of nodes in the network. As the number of possible graphs exponentially increases with the sizes of the networks, estimation of *θ*_Q_ and their standard errors rely on Markov Chain Monte Carlo (MCMC) simulations, as do the goodness of fit (GOF) tests of ERGMs. MPNet software was used to conduct the analyses which implements the MCMC maximum-likelihood estimation algorithm for ERGMs^30^.

A separate model was fitted for each of the two community multilevel networks. Based on the common ERGM specifications and the model goodness of fit (GOF) guided model selection strategy, that is, parsimonious models that can adequately reproduce the overall multilevel structure while respect the hierarchy of tie-dependence, we derived the final model specification for the two communities^31^. We test the GOF of each fitted models by simulating 5 thousand graphs from 50 million MCMC simulation updates using MPNet. The model GOF test procedures and results are presented in the supplementary material following the simulated based ERGM GOF test method^32^. The possible interpretations of selected model configurations or network effects are listed in Fig. 3 in the context of collaborative processes in community-based interventions.

We modelled a number of within level network effects for collaboration network (*A*) such as reciprocity and covariate match in committee members’ level of education and their organization affiliations. We also modelled in- and out-degree centralizations (AinS, AoutS), their correlation (AinAoutS) and network closure structures (ATT, ACT). For the meso-level choices of actions (*X*), we modelled the presence of active committee members (ASA), where steering committee members direct action towards many variables in the CLD, and for the presence of popular or key variables in the CLD that were more selected than others (ASB). Finally, we were interested in cross level triangles, 3-path and 4-cycle structures (TXBX, L3AXB and C4entrianment). We were particularly interested in cross-level closure configuration (C4entrainment) as it may be associated with the community’s ability to allocate and share intervention resources, to create integrated solutions and collective action, and ultimately achieve more effective intervention outcomes^24^.

## Results

**Figure 4:**
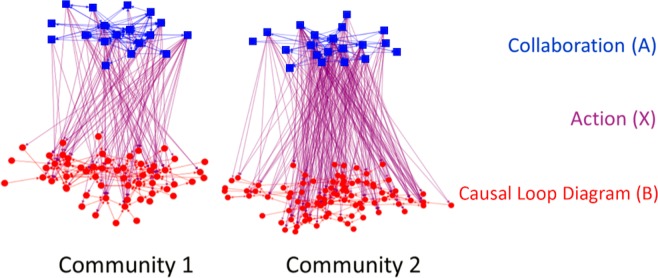
Visualization of multilevel network data for Community 1 and Community 2.

Community 1 had 18 respondents of 27 invitees (67%) and Community 2 had 20 respondents of 27 invitees (74%). Participants’ organizational affiliations and education level are shown in Table 1, which served as categorical attributes for community members in the ERGMs. On average, steering committee members in Community 1 nominated seven collaborative relationships (min 0, max 15), and Community 2 members made average of six nominations (min 0, max 17). Two MERGMs are fitted to test the dependencies among the steering committee collaboration network (A), the CLD (B) and committee members’ actions towards variables in the CLD (X). Figure 4 presents visualizations of each multilevel network.

**Table 1:** Descriptive statistics for participants, their actions and the CLD.

	Community 1	Community 2
**Collaboration Network**		
Number of respondents (n)	18	20
Female (n,%)	14 (78%)	18 (90%)
** Organisational affiliation (n,%)**		
Local Government	4 (22%)	3 (15%)
Education	3 (17%)	3 (15%)
Health Service	4 (22%)	3 (15%)
Primary Care Partnership	5 (28%)	1 (5%)
Other	2 (11%)	10 (50%)
** Education level (n,%)**		
Less than Year 12	0 (0%)	1 (5%)
Year 12 or equivalent	1 (6%)	0 (0%)
Diploma or Advanced Diploma	2 (11%)	5 (25%)
Bachelor’s Degree	9 (50%)	7 (35%)
Graduate Certificate or Graduate Diploma	4 (22%)	6 (30%)
Master’s Degree	2 (11%)	1 (5%)
**Average out-degree (collaboration) (mean, range)**	7.1 (min 0, max 15)	6.0 (min 0, max 17)
**CLD Network**		
CLD variables (n)	58	64
Average actions (from committee members to CLD variables) (mean, range)	5.4 (min 0, max 17)	10.7 (min 0, max 29)

The parameter estimates for the MERGMs are shown in Table 2, where a parameter is considered significant and marked by ‘*’, if the ratio between the estimates and estimated standard errors are greater than 2.0 in absolute value. Non-significant effects are included in order to provide adequate fit to the overall network structure. Empty cells mean the effects are not necessary to be included to provide adequate fit and can be considered as 0s. We present the interpretations of the model effects in the orders of collaboration network (A), meso-level choice of actions (X), and the interaction effects among collaboration, choice of actions and CLDs.

**Table 2:** Multilevel ERGM (MERGM) parameter estimates.

	Effects	Community 1			Community 2		
		Est.	Std. Err.		Est.	Std. Err.	
Collaboration (A)	Density	4.296	3.856		3.401	4.174	
	Reciprocity	1.362	0.481	*	2.488	0.564	*
	In2Star	0.127	0.057	*	0.159	0.044	*
	AinS	−3.153	1.201	*	−2.781	1.244	*
	AoutS	−1.404	0.943		−0.992	0.982	
	AinAoutS	−2.306	1.057	*	−1.837	1.131	
	ATT	1.029	0.304	*	0.956	0.258	*
	ACT				−0.455	0.168	*
	Edu_Match	0.141	0.213		0.041	0.254	
	Org_Match	0.947	0.304	*	−0.510	0.300	
Choice of action (X)	Density	−5.310	0.456	*	−5.798	0.577	*
	ASA	1.031	0.267	*	1.667	0.296	*
	ASB	0.789	0.188	*			
	ACA				0.069	0.008	*
A and X Interaction	ATXAX				0.152	0.142	
B and X interaction	In2StarBX				0.033	0.070	
	Out2StarBX				−0.117	0.072	
	TXBX	0.433	0.089	*	−0.107	0.163	
	L3XBXreciprocity				−1.011	0.880	
Cross level interaction	L3AXBin	0.021	0.006	*			
	C4AXBentrainment	−0.054	0.018	*	0.061	0.030	*
	C4AXBexchange				−0.044	0.031	
	C4AXBreciprocity				1.025	1.253	

### Collaboration (network A)

In both communities, collaboration ties (network A) tended to be reciprocated (based on significant positive Reciprocity parameter), meaning that committee members tended to agree on or confirm their collaborations with each other. The significant and positive ‘In2Star’ effects in both models indicate that collaborations tend to be centralized on a few committee members, that is, there are some popular members who received a higher portion of incoming collaborative ties relative to other members. However, there was also a ceiling effect on member popularity (negative AinS), meaning there are cost associated in maintaining many collaborations, and the networks are not extremely centralized. The model for Community 1’s steering committee had a significant negative correlation between collaboration in- and out-degree (AinAoutS), suggesting that those who received more incoming ties (high in-degree) were less likely to nominate other collaborators (low out-degree); while those who nominated more collaborators (high out-degree) tended to receive fewer nominations (low in-degree). This effect was not found in Community 2. Collaborations in both communities were more likely to form transitive closures (positive ATT), meaning that steering committee members tended to collaborate in groups where their direct collaborators also collaborated with one another. Furthermore, in Community 2, there was a tendency against generalized exchange (negative ACT). Combined with the positive ATT, the collaboration network in Community 2 tended to be more hierarchical.

Effects that tested for the role of node attributes found evidence for homophily in the collaboration network, indicating that committee members had a preference to have collaboration ties to others with similar attributes. In the model for Community 1, committee members were more likely to have a collaboration tie with individuals within the same organization type (Org_Match), than individuals who were in different organization types. In Community 2, this homophily effect is negative (albeit not statistically significant).

### Choice of actions in the system (network X)

When the participants were asked to identify their actions toward obesity driver variables in the CLD, Community 1 participants worked on an average of five variables (min. = 0, max. = 17) while community 2 participants worked on twice that number (average = 11, min. = 0, max. = 29).

The significant MERGM effects predicting committee members’ actions towards CLD variables (network X) can be interpreted as follows: for both communities, the positive, significant ASA effects indicate that the community steering committee network had some highly active members, who work on a high number of variables in the CLD. In Community 1, there is evidence that some CLD variables are selected more than others; indicating that there were some “popular” key obesity drivers, evident by the positive ASB effect. In Community 2, the positive ACA effect suggests key variables are also present in the CLD, and are chosen by sets of common committee members.

### Interactions between collaboration (A) and choice of action (X), or CLD (B) and action (X)

There is a positive and significant triangle TXBX effect in Community 1, which suggests that steering committee members were more likely to act upon variables that are connected in the CLD (network B). Other forms of interactions (e.g. ATXAX, In- or Out2StarBX and L3XBX reciprocity) are not significant, but important to include in the model for adequacy in goodness of fit tests.

### Cross-level interactions in the system (dependencies between network A, X and B)

Community 1 had a positive and significant L3AXBin effect, which provides two important interpretations. Firstly, this shows popular steering committee members in the collaboration network (those with high in-degrees) tend to work on variables that are also the most central (high in-degree) in the CLD. Secondly, this structure also represents a precondition or opportunity to form cross-level closures. Coupling this effect with the negative, significant C4AXBentrainment effect, where collaborating members work on connected variables in the CLD, shows the lack of, or a potential strengthening opportunity for cross-level closure in Community 1.

In Community 2, the positive and significant C4AXBentrainment parameter suggests that steering committee members were more likely to collaborate with other steering committee members who work on CLD variables that are connected to the CLD variables they are acting upon. In other words, in Community 2, an individual who works on a certain CLD variable is also more likely to collaborate with another steering committee member who is working on the ‘up-stream’ factor in the CLD.

## Discussion

We sought to explore the application of multilevel network analysis to bridge two complex components of obesity intervention evaluation: causal loop diagrams (CLDs) and steering committee networks, which previously been considered in isolation. We hypothesized there were likely interdependencies between these components that could improve our understanding of complex systems interventions. Multilevel exponential random graph models were used to uncover the patterns and structures of the formation of collaborative ties around the local complex systems of obesity drivers.

Based on the two models, the steering committees seemed to share a number of common properties with regards to the structure of their collaboration ties, including reciprocated ties, transitive collaboration and some evidence of centralization. Social network theory and literature suggest that networks with high centralization (though not overly centralized) can facilitate faster diffusion of ideas and innovations, and are expected to adopt programs more rapidly^17^. Furthermore, high reciprocity is seen to facilitate higher levels of trust^33^, which each appear ideal properties for these interventions.

The significant positive transitivity property in both steering committees is indicative of multiple two-paths leading to the formation of a direct tie. This is an efficient collaborative structure as it indicates a ‘path shortening’ pattern of collaboration between steering committee members^34^. For example, if a steering committee member seeks advice through an individual *i*, who is required to ask another individual *j*, the initial member will eventually seek the advice directly from *j*. While both communities displayed transitive collaboration patterns, Community 2 coupled this with a negative significant cyclic pattern. This shows local hierarchical structures being formed in Community 2^26^.

The negative significant correlation between in and out degree in Community 1’s steering committee networks means members who received more collaboration ties are less likely to send them, representing steering committee members have differing roles as senders or receivers of collaborative ties^26^. This could represent varying levels of influence or experience in the steering committee, whereby more experienced members are more popular receivers of collaborative nominations, but less active and perhaps more selective in sending and actively forming collaborations with others.

Community 1 steering committee members worked on less variables on average than Community 2 and tended to work on variables that are connected in the CLD, meaning they prefer to work on two variables that have a causal relationship. Community 1 were likely to form a collaboration tie within their organization, which was not significant in Community 2, meaning that steering committee members in Community 2 might not favour collaboration with others from the same organization type as their own organizations, but reach out to organizations from a different sector. Many of these differing model components could be potentially attributed to Community 2 being further into the intervention, though this requires further investigation.

We had expected to find positive cross-level entrainment in both communities, as other studies have identified cross-level structures to be associated with more robust performance due to increased sharing of resources and experiences^24^. Community 2 had significant positive effect of cross-level closure showing steering committee members collaborate when the variables they are working on are connected. For example, committee members are more likely to form a collaboration tie with someone who works in an area of the causal loop diagram that influences the variable they are working on. However, it was a negative significant effect in Community 1, coupled with the positive L3AXBin (a precondition to cross-level closure). This presents a potential opportunity to strengthen collaboration in Community 1, however given their shorter time in the intervention, it is possible that the cross-level effect will become ‘closed’ in Community 1, but longitudinal data is needed to confirm this.

From a multilevel network modelling perspective, we found that a within level ERGM for the collaboration network (A) for Community 1 would not converge without the inclusion of both (B) and (X). In other words, the network structure of collaboration cannot be modelled by ERGMs without the understanding of the structures of the choices of actions by the steering committee members, as well as the structure of the CLD. This further highlights the complexity of community-based systems interventions and how complex structures at one level may be explained by structures at a different level.

This study represents one of the first contributions of MERGMs to the public health literature and to our knowledge the first of a community-based systems intervention for obesity prevention. The results explain how collaboration of leaders is distributed around a complex whole-of-systems intervention, and may provide useful insight into bridging new ties to strength cross-level collaboration and action. Prior to this study, network analysis had been applied to causal loop diagrams and the collaboration of steering committees, but only in isolation of each other.

The study comprised only two communities that were studied cross-sectionally. This means the results may not be generalizable elsewhere, and causal relationships between multilevel network structure and intervention outcome cannot be identified. However, given these interventions have had high engagement, the results may provide insight for future intervention designs. A further limitation of this study results from the two MERGMs being developed independent of each other requiring us to compare the results from the two communities qualitatively.

The final models were selected using a GOF guided strategy for ERGM, aiming at a parsimonious model that captures not only the modelled effects, but other graph statistics in addition. The models provide adequate fits to all graph statistics implemented in the MPNet software. While this is a data-driven approach, future work could explore the inclusion of additional motifs that may be of interest to community stakeholders. For example, additional structures that are thought to be problematic or beneficial to the community-based intervention, based on qualitative feedback from community leaders, could be explored in future modelling processes.

Future work in this area should seek to further understand the implication of the findings for intervention designs and how alterations to the networks may be applied to strengthen current efforts. Heterogeneity in intervention outcomes such as engagement, changes in obesity prevalence or behaviours could allow our hypotheses regarding effectiveness of multilevel substructures to be tested and confirmed. Applying these techniques to future obesity interventions, along with interventions from other areas of public health, could allow complexities to be unpacked in diverse areas.

## Conclusion

The application of multilevel network analysis allows a deeper exploration of complex processes in community-based obesity prevention interventions. Multilevel network analysis in this context may assist in providing a more sophisticated understanding of the complex nature of public health systems interventions. The network models in this study suggested some similar and different collaborative formations, and opportunities for strengthening networks were observed. Exploration of more communities, along with the collection of longitudinal data, will assist with further unpacking the complexities involved in community-based obesity prevention interventions and their impact on intervention success.

## Supplementary information

Supplementary Table
