# Supplementary material for: Collaboration in Complex Systems: Multilevel Network Analysis for Community-Based Obesity Prevention Interventions
Source: Sci Rep. 2019 Aug 29;9:12599. doi: 10.1038/s41598-019-47759-4 (PMC6715639; doi:10.1038/s41598-019-47759-4)
Supplement: Supplementary file 1 — Supplementary Table [file 41598_2019_47759_MOESM1_ESM.docx]

**Collaboration in Complex Systems: Multilevel Network Analysis for Community-Based Obesity Prevention Interventions**

Jaimie McGlashan*^1^, Kayla de la Haye^2^, Peng Wang^3^, Steven Allender^1^

^1^ Global Obesity Centre, Deakin University, Geelong, Australia

^2^ Department of Preventive Medicine, University of Southern California, Los Angeles, California, United States of America

^3^ Centre for Transformative Innovation, Swinburne University of Technology, Melbourne, Australia

|  | Community 1 | | | | Community 2 | | | |
| --- | --- | --- | --- | --- | --- | --- | --- | --- |
| Statistics | Observed | Mean | StdDev | t-ratio | Obs. | Mean | StdDev | t-ratio |
| ArcA | 91 | 90.087 | 44.101 | 0.021 | 86 | 85.516 | 42.107 | 0.011 |
| ReciprocityA | 29 | 28.683 | 16.373 | 0.019 | 26 | 25.651 | 17.053 | 0.020 |
| In2StarA | 299 | 292.182 | 233.636 | 0.029 | 265 | 258.626 | 273.199 | 0.023 |
| Out2StarA | 310 | 303.701 | 214.499 | 0.029 | 272 | 263.597 | 224.643 | 0.037 |
| In3StarA | 737 | 734.016 | 713.475 | 0.004 | 767 | 703.107 | 1030.240 | 0.062 |
| Out3StarA | 736 | 726.527 | 585.762 | 0.016 | 636 | 620.270 | 685.893 | 0.023 |
| TwoPathA | 556 | 578.579 | 443.329 | -0.051 | 487 | 481.539 | 475.515 | 0.011 |
| Transitive-TriadA | 320 | 304.603 | 253.156 | 0.061 | 195 | 232.335 | 290.199 | -0.129 |
| Cyclic-TriadA | 91 | 95.464 | 83.031 | -0.054 | 49 | 63.271 | 89.037 | -0.160 |
| T1A | 21 | 19.841 | 17.654 | 0.066 | 11 | 16.672 | 25.763 | -0.220 |
| T2A | 166 | 158.712 | 139.007 | 0.052 | 88 | 124.622 | 185.292 | -0.198 |
| T3A | 215 | 212.574 | 184.944 | 0.013 | 115 | 154.151 | 222.348 | -0.176 |
| T4A | 118 | 109.278 | 94.852 | 0.092 | 65 | 86.021 | 120.873 | -0.174 |
| T5A | 115 | 110.540 | 91.804 | 0.049 | 71 | 85.500 | 111.486 | -0.130 |
| T6A | 131 | 138.748 | 118.360 | -0.065 | 107 | 117.307 | 148.826 | -0.069 |
| T7A | 391 | 395.843 | 331.975 | -0.015 | 329 | 336.364 | 402.056 | -0.018 |
| T8A | 390 | 403.936 | 313.651 | -0.044 | 338 | 336.755 | 354.295 | 0.004 |
| SinkA | 2 | 1.761 | 1.580 | 0.151 | 2 | 2.377 | 1.631 | -0.231 |
| SourceA | 0 | 0.172 | 0.437 | -0.393 | 1 | 0.298 | 0.548 | 1.281 |
| IsolateA | 0 | 0.002 | 0.040 | -0.040 | 0 | 0.003 | 0.056 | -0.057 |
| AinSA | 120.714 | 118.903 | 79.370 | 0.023 | 106.220 | 105.198 | 78.704 | 0.013 |
| AoutSA | 125.443 | 123.788 | 77.414 | 0.021 | 112.029 | 111.262 | 75.106 | 0.010 |
| AinSA2 | 120.714 | 118.903 | 79.370 | 0.023 | 106.220 | 105.198 | 78.704 | 0.013 |
| AoutSA2 | 125.443 | 123.788 | 77.414 | 0.021 | 112.029 | 111.262 | 75.106 | 0.010 |
| AinAoutSA | 47.593 | 47.375 | 18.806 | 0.012 | 47.751 | 47.628 | 14.347 | 0.009 |
| ATA-T | 141.742 | 139.559 | 97.485 | 0.022 | 113.031 | 111.763 | 97.960 | 0.013 |
| ATA-C | 126.938 | 129.710 | 97.343 | -0.028 | 91.266 | 89.438 | 93.034 | 0.020 |
| ATA-D | 139.477 | 137.043 | 95.350 | 0.026 | 115.871 | 106.249 | 90.918 | 0.106 |
| ATA-U | 134.594 | 135.151 | 98.552 | -0.006 | 105.328 | 106.765 | 99.413 | -0.014 |
| ATA-TD | 281.219 | 276.602 | 192.803 | 0.024 | 228.902 | 218.012 | 188.773 | 0.058 |
| ATA-TU | 276.336 | 274.710 | 195.902 | 0.008 | 218.359 | 218.528 | 197.226 | -0.001 |
| ATA-DU | 274.070 | 272.193 | 193.693 | 0.010 | 221.199 | 213.014 | 190.004 | 0.043 |
| ATA-TDU | 415.813 | 411.752 | 291.136 | 0.014 | 334.231 | 324.777 | 287.906 | 0.033 |
| A2PA-T | 314.336 | 296.663 | 186.127 | 0.095 | 345.063 | 275.728 | 179.625 | 0.386 |
| A2PA-D | 169.887 | 158.859 | 89.472 | 0.123 | 186.561 | 153.452 | 80.636 | 0.411 |
| A2PA-U | 159.098 | 142.742 | 97.143 | 0.168 | 183.289 | 138.066 | 102.241 | 0.442 |
| A2PA-TD | 484.223 | 455.522 | 274.410 | 0.105 | 531.623 | 429.180 | 258.345 | 0.397 |
| A2PA-TU | 473.434 | 439.405 | 282.614 | 0.120 | 528.352 | 413.794 | 280.776 | 0.408 |
| A2PA-DU | 328.984 | 301.601 | 184.023 | 0.149 | 369.850 | 291.519 | 178.843 | 0.438 |
| A2PA-TDU | 643.320 | 598.264 | 370.069 | 0.122 | 714.912 | 567.246 | 358.247 | 0.412 |
| Edu_MatchA | 29 | 28.775 | 14.731 | 0.015 | 23 | 22.863 | 11.908 | 0.012 |
| Edu_MismatchA | 62 | 61.312 | 30.621 | 0.022 | 63 | 62.653 | 31.131 | 0.011 |
| Edu_MatchReciprocityA | 9 | 9.486 | 5.911 | -0.082 | 6 | 7.103 | 5.053 | -0.218 |
| Edu_MismatchReciprocityA | 20 | 19.197 | 11.318 | 0.071 | 20 | 18.548 | 12.544 | 0.116 |
| Org_MatchA | 24 | 24.053 | 8.185 | -0.006 | 13 | 12.777 | 7.387 | 0.030 |
| Org_MismatchA | 67 | 66.034 | 37.076 | 0.026 | 73 | 72.739 | 35.982 | 0.007 |
| Org_MatchReciprocityA | 10 | 9.373 | 3.993 | 0.157 | 3 | 2.985 | 2.649 | 0.006 |
| Org_MismatchReciprocityA | 19 | 19.310 | 13.172 | -0.024 | 23 | 22.666 | 15.017 | 0.022 |
| XEdge | 97 | 99.186 | 27.946 | -0.078 | 214 | 213.459 | 41.320 | 0.013 |
| XStar2A | 373 | 360.792 | 169.575 | 0.072 | 1540 | 1529.391 | 579.947 | 0.018 |
| XStar2B | 152 | 139.612 | 62.616 | 0.198 | 230 | 258.811 | 102.976 | -0.280 |
| XStar3A | 1152 | 969.390 | 680.461 | 0.268 | 8705 | 9956.367 | 11627.069 | -0.108 |
| XStar3B | 210 | 153.371 | 96.699 | 0.586 | 151 | 203.401 | 127.668 | -0.410 |
| X3Path | 2005 | 2059.058 | 1307.608 | -0.041 | 6629 | 7232.047 | 3742.152 | -0.161 |
| X4Cycle | 104 | 114.461 | 92.134 | -0.114 | 265 | 283.335 | 187.199 | -0.098 |
| XECA | 1408 | 1883.029 | 2076.092 | -0.229 | 8726 | 9478.293 | 8575.606 | -0.088 |
| XECB | 775 | 754.502 | 759.471 | 0.027 | 961 | 1323.156 | 1187.153 | -0.305 |
| IsolatesXA | 2 | 2.110 | 1.933 | -0.057 | 2 | 1.773 | 1.512 | 0.150 |
| IsolatesXB | 18 | 20.614 | 6.467 | -0.404 | 3 | 6.842 | 4.075 | -0.943 |
| XASA | 136.363 | 140.234 | 48.653 | -0.080 | 356.813 | 355.756 | 77.729 | 0.014 |
| XASB | 84.750 | 87.289 | 34.235 | -0.074 | 169.844 | 180.767 | 59.396 | -0.184 |
| XACA | 328.625 | 311.275 | 135.344 | 0.128 | 1412.250 | 1395.790 | 507.581 | 0.032 |
| XACB | 109.375 | 97.540 | 36.362 | 0.325 | 144.016 | 163.408 | 51.264 | -0.378 |
| XAECA | 390.428 | 444.075 | 366.005 | -0.147 | 1057.203 | 1128.779 | 748.391 | -0.096 |
| XAECB | 343.594 | 361.211 | 318.397 | -0.055 | 651.000 | 784.519 | 608.119 | -0.220 |
| In2StarAX | 489 | 519.253 | 271.310 | -0.112 | 1205 | 1115.085 | 759.893 | 0.118 |
| Out2StarAX | 466 | 465.035 | 252.445 | 0.004 | 1236 | 1131.652 | 720.201 | 0.145 |
| AXS1Ain | 674.859 | 738.302 | 396.973 | -0.160 | 2094.984 | 1909.410 | 1347.847 | 0.138 |
| AXS1Aout | 654.832 | 642.059 | 361.512 | 0.035 | 2129.606 | 1939.288 | 1271.043 | 0.150 |
| AAinS1X | 655.386 | 697.248 | 480.567 | -0.087 | 1664.204 | 1497.517 | 1335.037 | 0.125 |
| AAoutS1X | 616.445 | 635.578 | 431.779 | -0.044 | 1710.672 | 1574.228 | 1224.131 | 0.111 |
| TXAXarc | 113 | 80.245 | 53.641 | 0.611 | 197 | 177.013 | 144.700 | 0.138 |
| TXAXreciprocity | 41 | 25.925 | 20.453 | 0.737 | 78 | 60.112 | 58.057 | 0.308 |
| ATXAXarc | 77.250 | 57.976 | 36.754 | 0.524 | 100.656 | 99.645 | 72.057 | 0.014 |
| ATXAXreciprocity | 27.125 | 18.764 | 13.919 | 0.601 | 36.703 | 32.506 | 28.330 | 0.148 |
| L3XAX | 2395 | 2627.719 | 1607.101 | -0.145 | 16238 | 14806.613 | 12284.114 | 0.117 |
| L3XAXreciprocity | 852 | 828.724 | 594.215 | 0.039 | 5920 | 5018.191 | 4907.237 | 0.184 |
| In2StarBX | 348 | 344.437 | 98.809 | 0.036 | 387 | 385.240 | 79.244 | 0.022 |
| Out2StarBX | 164 | 207.688 | 74.075 | -0.590 | 340 | 338.768 | 73.397 | 0.017 |
| AXS1Bin | 403.313 | 374.886 | 144.389 | 0.197 | 318.438 | 333.500 | 116.079 | -0.130 |
| AXS1Bout | 107.953 | 183.360 | 94.246 | -0.800 | 247.813 | 268.277 | 100.240 | -0.204 |
| ABinS1X | 445.641 | 434.279 | 129.195 | 0.088 | 255.438 | 251.559 | 56.023 | 0.069 |
| ABoutS1X | 120.266 | 176.566 | 77.578 | -0.726 | 212.750 | 216.179 | 53.910 | -0.064 |
| TXBXarc | 56 | 58.547 | 29.656 | -0.086 | 56 | 54.980 | 24.099 | 0.042 |
| TXBXreciprocity | 1 | 1.262 | 1.412 | -0.186 | 0 | 0.115 | 0.350 | -0.329 |
| ATXBXarc | 46.625 | 46.309 | 20.287 | 0.016 | 50.250 | 50.626 | 21.354 | -0.018 |
| ATXBXreciprocity | 1 | 0.960 | 0.914 | 0.043 | 0 | 0.114 | 0.344 | -0.331 |
| L3XBX | 523 | 658.730 | 367.079 | -0.370 | 886 | 896.683 | 385.694 | -0.028 |
| L3XBXreciprocity | 4 | 11.432 | 13.091 | -0.568 | 2 | 1.881 | 2.364 | 0.050 |
| L3AXBin | 1970 | 1960.176 | 1161.435 | 0.008 | 2108 | 2046.024 | 1434.240 | 0.043 |
| L3AXBout | 647 | 878.160 | 436.456 | -0.530 | 1960 | 1843.208 | 1201.741 | 0.097 |
| L3AXBpath | 697 | 1018.107 | 483.382 | -0.664 | 1870 | 1774.605 | 1242.134 | 0.077 |
| L3BXApath | 1864 | 1742.170 | 1067.323 | 0.114 | 2185 | 2042.197 | 1336.589 | 0.107 |
| C4AXBentrainment | 292 | 292.095 | 179.009 | -0.001 | 624 | 611.983 | 524.191 | 0.023 |
| C4AXBexchange | 320 | 310.137 | 186.862 | 0.053 | 572 | 559.959 | 491.554 | 0.024 |
| C4AXBexchangeAreciprocity | 220 | 186.947 | 134.652 | 0.245 | 422 | 400.834 | 405.773 | 0.052 |
| C4AXBexchangeBreciprocity | 0 | 7.587 | 9.539 | -0.795 | 4 | 4.308 | 6.997 | -0.044 |
| C4AXBreciprocity | 0 | 1.083 | 1.662 | -0.652 | 1 | 0.953 | 1.645 | 0.029 |
| AinASXAinBS | 1101.026 | 1131.527 | 558.607 | -0.055 | 0 | 91.154 | 762.586 | -0.120 |
| AoutASXAoutBS | 736.711 | 812.144 | 415.697 | -0.181 | 1923.422 | 1790.406 | 1269.320 | 0.105 |
| AinASXAoutBS | 775.651 | 873.814 | 463.666 | -0.212 | 1876.954 | 1713.695 | 1380.011 | 0.118 |
| AoutASXAinBS | 1062.086 | 1069.857 | 510.750 | -0.015 | 1966.109 | 1825.787 | 1271.797 | 0.110 |
| stddev_indegreeA | 3.670 | 2.379 | 1.066 | 1.211 | 3.600 | 2.497 | 1.256 | 0.878 |
| skew_indegreeA | 0.572 | 0.444 | 0.466 | 0.274 | 1.621 | 0.684 | 0.559 | 1.677 |
| stddev_outdegreeA | 3.842 | 2.686 | 0.967 | 1.196 | 3.701 | 2.780 | 0.782 | 1.178 |
| skew_outdegreeA | 0.230 | 0.274 | 0.600 | -0.072 | 0.579 | 0.534 | 0.561 | 0.080 |
| clusteringA_tm | 0.576 | 0.453 | 0.128 | 0.961 | 0.400 | 0.379 | 0.122 | 0.176 |
| clusteringA_cm | 0.491 | 0.395 | 0.156 | 0.615 | 0.302 | 0.265 | 0.145 | 0.255 |
| clusteringA_ti | 0.535 | 0.466 | 0.127 | 0.544 | 0.368 | 0.373 | 0.105 | -0.051 |
| clusteringA_to | 0.516 | 0.409 | 0.149 | 0.725 | 0.359 | 0.326 | 0.147 | 0.223 |
| stddev_degreeX_A | 4.341 | 3.603 | 0.773 | 0.954 | 7.270 | 6.551 | 2.216 | 0.325 |
| skew_degreeX_A | 0.851 | 0.289 | 0.431 | 1.303 | 0.644 | 0.452 | 0.861 | 0.223 |
| stddev_degreeX_B | 2.047 | 1.817 | 0.354 | 0.649 | 1.290 | 1.451 | 0.170 | -0.946 |
| skew_degreeX_B | 1.608 | 1.122 | 0.414 | 1.174 | 0.643 | 0.450 | 0.259 | 0.743 |
| clusteringX | 0.208 | 0.201 | 0.056 | 0.117 | 0.160 | 0.147 | 0.023 | 0.557 |
